# Elevated Serum IgG4 Defines Specific Clinical Phenotype of Rheumatoid Arthritis

**DOI:** 10.1155/2014/635293

**Published:** 2014-12-07

**Authors:** Le-Feng Chen, Ying-Qian Mo, Jian-Da Ma, Ling Luo, Dong-hui Zheng, Lie Dai

**Affiliations:** ^1^Department of Rheumatology, Sun Yat-Sen Memorial Hospital, Sun Yat-Sen University, No. 107 Yan Jiang West Road, Guangzhou 510120, China; ^2^Department of Clinical Laboratory, Sun Yat-Sen Memorial Hospital, Sun Yat-Sen University, No. 107 Yan Jiang West Road, Guangzhou 510120, China

## Abstract

*Objectives*. To explore the correlation of serum IgG4 (sIgG4) with clinical manifestations or therapeutic response in rheumatoid arthritis (RA).* Methods*. Consecutive 136 RA patients were recruited and followed up at regular interval. SIgG4 was detected by immunonephelometry. Serial synovial tissue sections from 46 RA patients were stained immunohistochemically for IgG4.* Results*. Forty-six percent of 136 RA patients had elevated sIgG4. Patients with elevated sIgG4 had higher sIgG4/sIgG ratio, C-reactive protein, erythrocyte sedimentation rate, rheumatoid factor, and anticyclic citrullinated peptide antibodies than those with normal sIgG4 (all *P* < 0.05). Among 45 patients who received methotrexate and leflunomide therapy, 50% (9/18) of patients with elevated sIgG4 and 85% (23/27) of patients with normal sIgG4 reached therapeutic target (disease activity score of 28 joints < 3.2) at 6-month visit (*χ* ^2^ = 6.508, *P* = 0.011). IgG4-positive plasma cell count correlated positively with sIgG4, total synovitis score, and CD3-, CD20-, and CD38-positive cell counts (all *P* < 0.05).* Conclusions*. Our results showed that elevated sIgG4 in RA is common and disproportional to total IgG and RA with elevated sIgG4 may be a specific clinical phenotype with higher disease activity, higher level of autoantibodies, and poor response to methotrexate and leflunomide therapy.

## 1. Introduction

Rheumatoid arthritis (RA) is a systemic autoimmune disease characterized by synovitis and joint destruction, leading to severe deformity and disability without proper therapy. RA is a heterogeneous disease. Previous studies showed that RA patients with anticyclic citrullinated peptide antibodies (anti-CCP Ab) had more swollen joints and more severe radiological destruction than those without anti-CCP Ab [1, 2]. Another study indicated that RA patients with high titer of anticollagen type II antibody may have a distinct clinical phenotype with significantly elevated C-reactive protein (CRP), erythrocyte sedimentation rate (ESR), TNF-*α*, IL1-*β*, and IL-8 at baseline [3]. Subtyping of RA may be helpful for optimal therapeutic strategies and outcome prediction.

Recently, much attention has been paid to IgG4 since the recognition of IgG4-related disease (IgG4-RD), a new emerging disease entity. IgG4-RD is a systemic disease characterized by swelling or masses in the involved organs, elevated serum IgG4 (sIgG4), and marked IgG4-positive plasma cells infiltration and fibrosis [4]. Elevation of serum and histological IgG4 separates Mikulicz's disease [5] from Sjögren syndrome and type 1 autoimmune pancreatitis [6] from autoimmune pancreatitis. Thus, elevation of IgG4 may define a specific clinical phenotype.

It was reported that sIgG4 elevated in RA patients compared to healthy control [7, 8]. However, the clinical significance of elevated IgG4 in RA remains elusive. Here we explored the correlation of IgG4 with clinical manifestations and therapeutic response in RA.

## 2. Materials and Methods

### 2.1. Patients

One hundred and thirty-six consecutive RA patients who fulfilled 1987 ACR revised classification criteria for RA or the 2010 ACR/EULAR classification criteria for early RA were recruited from April 2010 to January 2013 at Sun Yat-Sen Memorial Hospital, Sun Yat-Sen University, Guangzhou. Patients with allergic disorders, pemphigus, parasite infestations, Castleman's disease, Churg-Strauss syndrome, or IgG4-RD were excluded. The study was approved by the Medical Ethics Committee of Sun Yat-Sen Memorial Hospital and all patients signed informed consent.

### 2.2. Clinical Assessments

All patients were followed up at regular interval. Demographic characteristics, RA clinical assessments, and therapeutic regimens were collected at baseline and 1st, 3rd, and 6th months. RA clinical assessments include the core set of disease activity measures for RA recommended by ACR [9] and measurement of three autoantibodies: rheumatoid factor (RF, determined by nephelometry, Siemens Healthcare Diagnostics, Munich, Germany, normal range < 20 IU/mL), anti-CCP Ab (measured by ELISA, Aesku Diagnostics, Wendelsheim, Germany, normal range < 18 U/mL), and antinuclear antibody (ANA, measured by ELISA, Aesku Diagnostics, Wendelsheim, Germany, normal range < 1.00 S/CO value).

### 2.3. Measurement of Serums IgG and IgG4

Serum was collected from all RA patients at enrollment and stored at −80°C. SIgG and sIgG4 levels were determined by immunonephelometry with BN ProSpec System (Dade Behring, Deerfield, IL, USA) using the following kits: N AS IgG and N Latex IgG4 (Siemens Healthcare Diagnostics Products GmbH, Marburg, Germany). SIgG > 16 g/L or sIgG4 ≥ 1.35 g/L was considered as elevated.

### 2.4. Synovial Tissues and Immunohistochemistry (IHC)

Closed Parker-Pearson needle synovial biopsy was performed on knees of 46 RA patients among the above patients at enrollment. At least 6 pieces of synovial tissue were obtained per patient to minimize sampling error [10]. All samples were immediately fixed in 10% neutral formalin and embedded in paraffin. Sections (5 *μ*m) were cut serially and mounted on adhesive glass slides. Sealed slides were stored at −20°C until staining.

Serial sections of synovial tissues were stained with hematoxylin and eosin and a 3-step immunoperoxidase method which was shown in detail in our previous study [11]. Serial sections were stained with rabbit anti-human IgG antibody (Cell Marque Corporation, California, USA), rabbit anti-IgG4 monoclonal antibody (clone EP138, Epitomics, Inc., Burlingame, USA), and the following commercial antibody preparations (Invitrogen, Carlsbad, CA, USA): anti-CD38 (clone SPC32, plasma cells), anti-CD3 (clone PS1, T cells), anti-CD20 (clone L26, B cell), anti-CD68 (clone KP1, macrophage-like synoviocytes and macrophages), and anti-CD34 (clone QBEnd/10, vascular endothelial cells). Tonsil tissues were used as positive control tissues in each staining run.

### 2.5. Assessment of Synovial IgG4-Positive Plasma Cells and Histological Synovitis

Only tissue pieces containing integrated synovial lining and sublining layer were included in the analyses. At least 3 qualified pieces were evaluated for each specimen. Histological synovitis was graded by two independent observers (Le-Feng Chen and Ying-Qian Mo) according to a three-component synovitis score [12] including enlargement of synovial lining layer, density of sublining resident cells, and inflammatory infiltration. Each feature was scored from 0 to 3 and the sum provided the synovitis score from 0 to 9.

The densities of sublining IgG- or IgG4-positive plasma cells were evaluated under microscope and photographed with the matching microscope imaging software (Leica DM2500, LAS V3.6, Leica Microsystems GmbH, Wetzlar, Germany). Since each photo revealed a synovial area of 0.11740 mm^2^, the densities of IgG4- or IgG-positive plasma cells were counted manually in 9 photos with total area of nearly 1 mm^2^ by two independent observers (Le-Feng Chen and Jian-Da Ma) and the results were showed as cells per mm^2^. The densities of CD38, CD3, CD20, CD68 positive-staining cells, or CD34-positive vessels were also counted as mentioned above and showed as cells or vessels per mm^2^.

### 2.6. Statistical Analysis

Statistical analysis was performed with SPSS for Windows 13.0 (SPSS Inc., Chicago, IL, USA). Data were described with mean ± standard deviation (range) or number (percentage) unless stated otherwise. Mann-Whitney rank-sum test was used for comparison between two groups and Kruskal-Wallis one-way analysis of variance on ranks among three or more groups. Spearman's rank order correlation test was used to assess the correlation between two variables. *P* < 0.05 was considered statistically significant unless stated otherwise.

## 3. Results

### 3.1. Characteristics of the Study Patients and Their sIgG4 Level

Demographic characteristics of 136 RA patients are shown in Table 1. None of the patients had an overlap of IgG4-RD with RA. Among 136 RA patients, the mean of sIgG4 was 1.52 ± 1.27 g/L (range 0.04~5.92 g/L) and 46% had elevated sIgG4. Patients were then divided into elevated sIgG4 group (*n* = 62) and normal sIgG4 group (*n* = 74). SIgG4 level correlated positively with sIgG level (*r* = 0.424, *P* < 0.001). The mean sIgG4/sIgG ratio of 136 RA patients was 10% ± 7% (range 0.3%~37%). The mean sIgG4/sIgG ratio of elevated sIgG4 group was 16% ± 7%, which was significantly higher than that of normal sIgG4 group (5% ± 3%; *P* < 0.001). SIgG4/sIgG ratio > 8% was reported as elevated [13]. Fifty-one percent of the 136 RA patients had elevated sIgG4/sIgG ratio. Ninety-five percent (59/62) in elevated sIgG4 group and only 14% (10/74) in normal sIgG4 group had elevated sIgG4/sIgG ratio (*χ* ^2^ = 89.976, *P* < 0.001).

**Figure 1:**
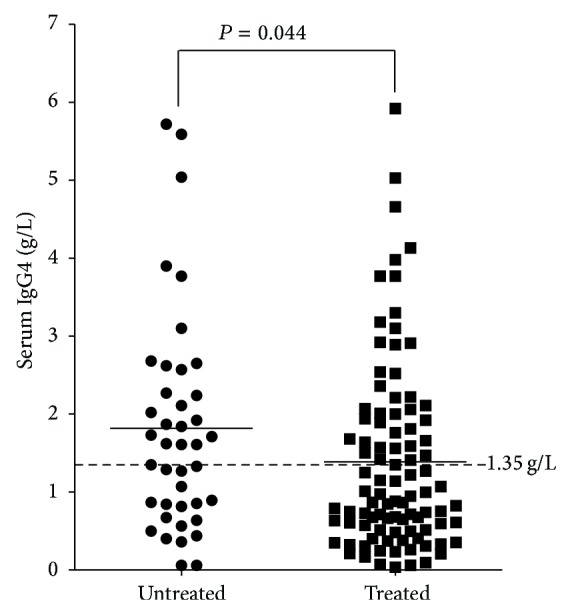
Comparison of serum IgG4 level between untreated and treated RA patients. Untreated RA patients (*n* = 41) had higher serum IgG4 level than that of treated RA patients (*n* = 95).

Thirty percent (41/136) of all RA patients had never taken any disease modifying antirheumatic drugs (DMARDs) or corticosteroid before enrollment, and demographic characteristics of these patients are showed in Table 1. The mean sIgG4 of the untreated patients was 1.82 ± 1.39 g/L, which was significantly higher than that of the treated patients (1.39 ± 1.20 g/L; *P* = 0.044, Figure 1). The mean sIgG4/sIgG ratio of the untreated patients was 11% ± 7% (range 0.4%~30%) and 63% had elevated sIgG4/sIgG ratio.

### 3.2. SIgG4 and Clinical Disease Activity

Among 136 RA patients, 16% were in disease remission (DAS28 < 2.6), 10% in low disease activity (LDA, DAS28 ≥ 2.6 to <3.2), 32% in moderate disease activity (MDA, DAS28 ≥ 3.2 to ≤5.1), and 41% in high disease activity (HDA, DAS28 > 5.1). The mean sIgG4 levels of patients in remission, LDA, MDA, or HDA groups were 0.94 ± 0.77 g/L, 1.38 ± 1.17 g/L, 1.54 ± 1.36 g/L, and 1.76 ± 1.33 g/L, respectively, which showed significant difference among these four groups (*χ* ^2^ = 8.456, *P* = 0.037). Mann-Whitney rank-sum test was used for comparison between two groups for 6 times since there were 6 pairs among four groups and *P* < 0.0083 (0.05/6) was considered statistically significant. Multiple comparisons revealed that sIgG4 of HDA group was significantly higher than that of remission group (*P* = 0.003). The ratios of patients with elevated sIgG4 in remission, LDA, MDA, or HDA groups were 32% (7/22), 43% (6/14), 43% (19/44), and 54% (30/56), respectively, showing no significant difference among these four groups (*P* > 0.05).

Patient global assessment of disease activity (PtGA), provider global assessment of disease activity (PrGA), CRP, ESR, and IgE of the elevated sIgG4 group were significantly higher than those of the normal sIgG4 group (PtGA, 6 ± 3 versus 4 ± 3; PrGA, 5 ± 3 versus 4 ± 3; CRP, 38 ± 42 mg/L versus 25 ± 33 mg/L; ESR, 70 ± 42 mm/h versus 48 ± 32 mm/h; IgE, 604 ± 1938 IU/mL versus 159 ± 275 IU/mL; all *P* < 0.05). SIgG4 level correlated positively but slightly with 28 tender joint counts (28TJC, *r* = 0.191), Health Assessment Questionnaire score (HAQ, *r* = 0.221), PtGA (*r* = 0.241), PrGA (*r* = 0.248), CRP (*r* = 0.373), ESR (*r* = 0.389), IgE (*r* = 0.328), and DAS28 (*r* = 0.253; all *P* < 0.05).

Among 41 untreated patients, no one was in disease remission, 7% were in LDA, 37% were in MDA, and 56% were in HDA. The mean sIgG4 levels of patients in LDA, MDA, or HDA groups were 1.41 ± 0.82 g/L, 1.86 ± 1.51 g/L, and 1.84 ± 1.41 g/L, respectively, showing no significant difference among these three groups (*P* > 0.05). CRP level of the elevated sIgG4 group (*n* = 23) was significantly higher than that of the normal sIgG4 group (*n* = 18; 44 ± 31 mg/L versus 29 ± 33 mg/L, *P* = 0.022). SIgG4 level correlated positively with CRP (*r* = 0.426) and ESR (*r* = 0.315; both *P* < 0.05).

### 3.3. SIgG4 and Autoantibodies

RF and anti-CCP Ab levels of the elevated sIgG4 group were significantly higher than those of the normal sIgG4 group (RF, 513 ± 636 IU/mL versus 245 ± 392 IU/mL; anti-CCP Ab, 256 ± 243 U/mL versus 162 ± 199 U/mL; both *P* < 0.05), but there was no significant difference of ANA between these two groups (1.2 ± 1.2 S/CO versus 1.1 ± 0.9 S/CO, *P* > 0.05). SIgG4 level correlated positively with RF (*r* = 0.355, *P* < 0.001) and anti-CCP Ab (*r* = 0.224, *P* = 0.009), but there was no significant correlation between sIgG4 and ANA (*P* > 0.05). The positive rate of RF in elevated sIgG4 group (89%) was significantly higher than that in normal sIgG4 group (73%; *χ* ^2^ = 5.250, *P* = 0.022), but there were no significant differences of positive rate of anti-CCP Ab or ANA between these two groups (both *P* > 0.05).

Seventy-five RA patients with active disease at baseline completed ≥6-month follow-up. RF and anti-CCP Ab levels of 67 patients were determined again at 6-month visit, and the results showed that RF and anti-CCP Ab levels significantly decreased after treatment both in elevated baseline sIgG4 group (*n* = 28) and in normal baseline sIgG4 group (*n* = 39, all *P* < 0.05). Reduction range of RF level after treatment in elevated baseline sIgG4 group was significantly larger than that in normal baseline sIgG4 group (*P* < 0.01, Table 2).

### 3.4. SIgG4 and Therapeutic Response

Among the 75 patients who completed ≥6-month follow-up, 45 patients received methotrexate (MTX) + leflunomide (LEF) therapy, 18 received MTX + tumor necrosis factor (TNF)-*α* antagonist (≥12 weeks), and 12 received other DMARD(s) therapies. For patients receiving MTX + LEF, 50% (9/18) of patients with elevated baseline sIgG4 were good responders who reached therapeutic target of DAS28 < 3.2 at 6-month visit, which was significantly lower than 85% (23/27) of patients with normal baseline sIgG4 (*χ* ^2^ = 6.508, *P* = 0.011). For patients receiving MTX + TNF-*α* antagonist, there was no significant difference in the percentage of good responders between patients with elevated baseline sIgG4 and normal baseline sIgG4, 70% (7/10) versus 88% (7/8), *P* > 0.05.

### 3.5. IgG4-Positive Plasma Cells in Synovium

**Figure 2:**
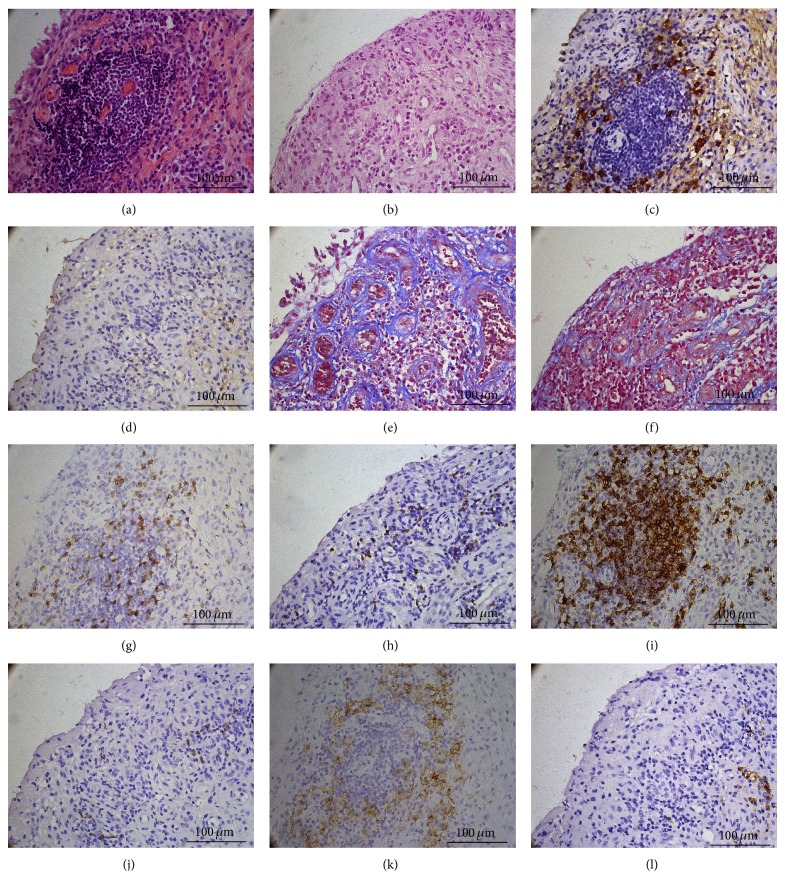
Histopathological features of synovium of 2 RA patients (400x). Images of the left column were from one RA patient with higher synovial IgG4-positive plasma cells and the right column from another RA patient with lower synovial IgG4-positive plasma cells. (a, b) H&E staining. (c, d) IgG4 immunohistochemistry staining. (e, f) Masson's trichrome staining. (g, h) CD3 immunohistochemistry staining. (i, j) CD20 immunohistochemistry staining. (k, l) CD38 immunohistochemistry staining.

Among 46 RA patients receiving synovial biopsy, 78% of them were female and the mean age was 55 ± 14 years, mean disease duration was 74 ± 75 months, and the mean DAS28 was 5.0 (range 1.2~8.2). As shown in Figure 2, cytoplasmic expression of IgG4 was extremely distributed in synovial sublining area. The IgG4-positive cells showed typical morphology of plasma cells and IHC staining of CD3, CD20, CD38, or CD68 on serial slides confirmed that IgG4 is mainly expressed in the CD38-positive plasma cells. The mean count of synovial IgG4-positive plasma cells was 155 ± 175/mm^2^ (range 0~735). IgG is also expressed in the sublining plasma cells with high background staining. IgG-positive cells were defined as clear-cut cells with strong cytoplasmic reactivity of IgG. The mean count of synovial IgG-positive plasma cells was 555 ± 474/mm^2^ (range 25~2020). The mean IgG4+/IgG+ plasma cell ratio was 26% ± 19% (range 0~82%).

**Figure 3:**
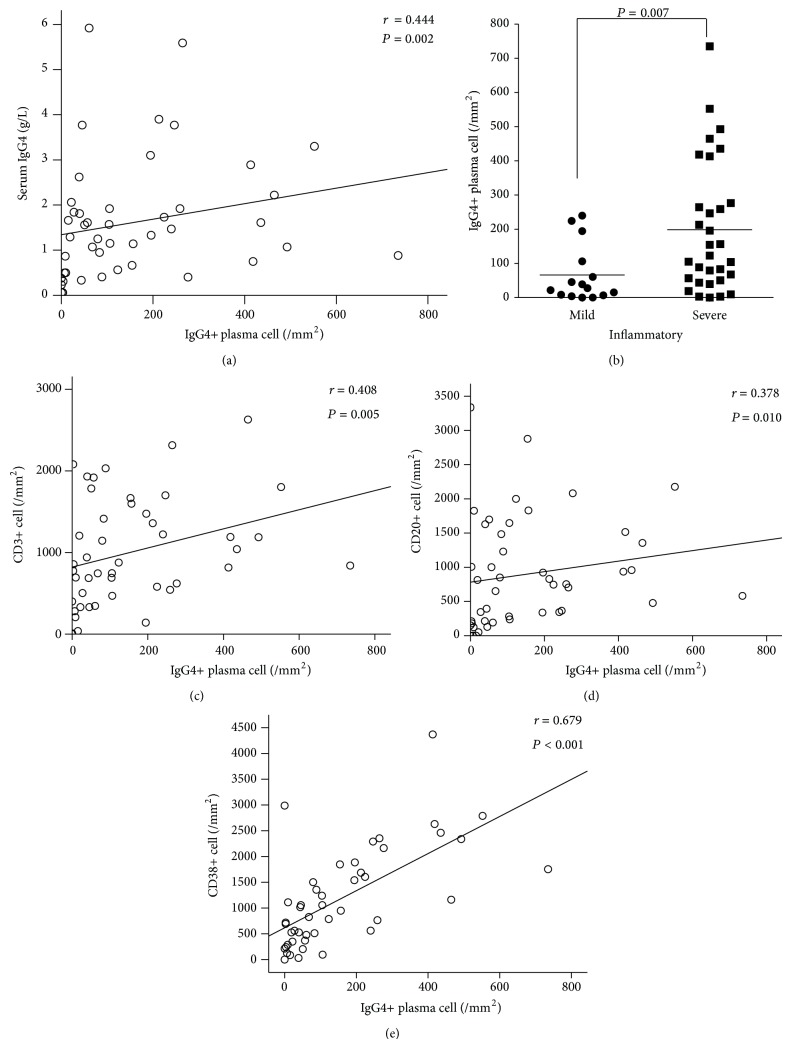
Correlation of IgG4-positive plasma cells with serum IgG4 and inflammatory infiltration in synovium of 46 RA patients. Spearman's rank order correlation test showed significant correlation of IgG4-positive plasma cells with serum IgG4 (a), CD3+ (c), CD20+ (d), and CD38+ cells (e). Patients with severe inflammatory infiltration (*n* = 31) had higher IgG4-positive plasma cells than those with mild inflammatory infiltration in synovium (*n* = 15) (b).

Spearman's rank order correlation test showed synovial IgG4-positive plasma cells correlated positively and significantly with total synovitis score (*r* = 0.374, *P* = 0.010), inflammatory infiltration subscore (*r* = 0.335, *P* = 0.023), and density of resident cells subscore (*r* = 0.364, *P* = 0.013). Patients were divided into severe inflammatory group (inflammatory infiltration subscore: 2~3, *n* = 31) and mild inflammatory group (0~1, *n* = 15). Patients in severe inflammatory group had higher IgG4-positive plasma cells than those in mild inflammatory group (202 ± 189/mm^2^ versus 49 ± 55/mm^2^, Figure 3(b)). Positive and significant correlation was found between IgG4-positive plasma cells and CD3-positive (Figure 3(c)), CD20-positive (Figure 3(d)), or CD38-positive cell counts (Figure 3(e)), but not CD68-positive cell count or CD34-positive vessel count (both *P* > 0.05).

SIgG4 correlated positively and significantly with synovial IgG4-positive plasma cells (Figure 3(a)), but no significant correlation of sIgG4 with total synovitis score or subscores was found.

## 4. Discussion

This study detected sIgG4 level of 136 RA patients and 46% of them had elevated sIgG4, which was disproportional to total sIgG. We first demonstrated that RA with elevated sIgG4 may be a specific clinical phenotype characterized by higher disease activity, higher level of autoantibodies, and poor response to MTX + LEF therapy. Further immune pathological study in synovium of 46 RA patients first demonstrated that elevated sIgG4 correlated with increased synovial IgG4-positive plasma cells, which correlated with histological synovitis in RA.

### 4.1. Elevated sIgG4 in RA

Elevated sIgG4 is not specific to IgG4-RD, although it is an important characteristic of IgG4-RD. Patients with allergic disorders, parasite infestations [14], pemphigus [15], Castleman's disease [16], or Churg-Strauss syndrome [8] may have elevated sIgG4. Recently, elevated sIgG4 was also reported in RA patients. Lin and Li [7] found that four subclasses of IgG (IgG1~IgG4) in serum were, respectively, significantly higher in 72 RA patients than that in healthy people, although sIgG4 remained the lowest subclasses. In this study, we found that 46% of RA patients had elevated sIgG4 determined by immunonephelometry.

The mechanism of IgG4 elevation seems different between RA and IgG4-RD. Synthesis of IgG4 in vitro was usually regulated by certain cytokines and chemokines such as IL-10 and IL-6 [17–19]. IL-10, a lymphokine with important anti-inflammatory property, enhanced IL-4-induced IgG4 switching. Serum IL-10 was elevated in IgG4-related pancreatitis, cholangitis [20], or tubulointerstitial nephritis [21], supporting that IL-10 might participate in the sIgG4 elevation and IgG4-positive plasma cell infiltration in IgG4-RD [20]. Serum IL-10 was also reported to be higher in RA patients compared to healthy controls [22], but the relationship between IL-10 and IgG4 elevation in RA remains elusive. IL-6 inducing IgG4 elevation might be partly through IL-21 expressed in CD4+ T cells [23], which promotes differentiation of B cells into antibody-secreting plasma cells [24]. Elevated IL-6 level has been found in serum or synovium of RA patients [25, 26], but not in IgG4-RD [27, 28]. IL-6 is a key proinflammatory cytokine in RA and plays important roles in the regulation of the immune response, inflammation, hematopoiesis, and bone metabolism [29]. SIgG4 decreased in 7 of 8 RA patients who received treatment of tocilizumab, a monoclonal antibody to the IL-6 receptor [30], indicating that IL-6 may be the principal cytokine that induces IgG4 elevation in RA.

SIgG4/sIgG ratio may be helpful in making diagnosis of IgG4-RD [4]. Eight percent were reported as cutoff point of sIgG4/sIgG ratio to discriminate IgG4-RD from other mimickers with specificity varying from 59% [31] to 87.5% [13]. In this study, we found that sIgG4/sIgG ratio of RA patients with normal sIgG4 group was 5% ± 3%, which was similar with 3%~7% reported in healthy people [32]. Our study also showed that sIgG4/sIgG ratio of RA patients with elevated sIgG4 group was 16% ± 7%, which was significantly higher than that of normal sIgG4 group, suggesting sIgG4 increased disproportionally to total sIgG in RA patients with elevated sIgG4.

### 4.2. Elevated sIgG4 May Define Specific Clinical Phenotype of RA

Autoantibodies such as RF, anti-CCP Ab, have IgG type which included IgG1~IgG4 subclasses. Cohen et al. [33] showed that RF-IgG1 was the most prevalent subclass of RF-IgG in RA patients, followed by RF-IgG4 which had higher level than RF-IgG2 or -IgG3. Similarly, anti-CCP Ab-IgG4 was also the second subclass of anti-CCP Ab-IgG in RA patients [34]. Our study demonstrated that RA patients with elevated sIgG4 had significantly higher RF and anti-CCP Ab levels at baseline, as well as higher reduction of RF after treatment, indicating that RA patients with elevated sIgG4 may have more IgG4 autoantibodies such as RF-IgG4 which was easy to decline during treatment. Despite relative lack of study on the change of RF-IgG subclasses including RF-IgG4, there were studies on the change of different anti-CCP Ab-IgG subclasses in RA patients during biological DMARDs therapy. Carbone et al. [30] reported that anti-CCP Ab-IgG4, but not anti-CCP Ab-IgG1, reduced significantly after 6-month tocilizumab treatment in RA patients. Bos et al. [35] reported that both anticitrullinated protein antibody- (ACPA-) IgG1 and IgG4 of RA patients reduced significantly after 46-week infliximab or 28-week adalimumab treatment, accompanied by significant reduction of ACPA-IgG4/IgG1 ratio, indicating preferential decrease in ACPA-IgG4. Our study did not find significant reduction of anti-CCP Ab between RA patients with elevated sIgG4 and normal sIgG4, perhaps due to different therapeutic regimes or predominant use of traditional DMARDs or the small number of patients.

For autoimmune pancreatitis, patients with elevated sIgG4 may be prone to systemic disease with high disease severity, and both sIgG4 and sIgG4/sIgG ratio significantly decreased after glucocorticoid therapy [36–38]. Similarly, elevated sIgG4 may correlate with disease activity in RA. Yamamoto et al. [8] demonstrated that 17% (5/29) of RA patients with active disease (DAS28: 5.1 ± 1.2) at onset had elevated sIgG4 level. In this study, we included 22 RA patients with disease remission and divided patients into different extents of disease activity to evaluate the correlation of sIgG4 with RA disease activity. Our result showed that sIgG4 of RA patients with HDA was significantly higher than those with disease remission. Further comparative analysis and correlation test confirmed that RA disease activity indexes including ESR, CRP, and DAS28 positively correlated with sIgG4. The untreated patients were analyzed separately and only positive correlation between sIgG4 and CRP or ESR was found. No correlation of sIgG4 with DAS28 in the untreated patients may be explained by bias of patient enrollment, since 93% of the untreated patients were in MDA or HDA. Our result also showed that only 50% of patients with elevated baseline sIgG4 reached DAS28 < 3.2 after 6-month MTX + LEF combination therapy, significantly lower than 85% of patients with normal baseline sIgG4. These results suggest that sIgG4 has correlation with clinical synovitis which determines clinical disease activity of RA patients.

### 4.3. Synovial IgG4-Positive Plasma Cells Correlate with Histological Synovitis

Marked IgG4-positive plasma cells infiltration and dense lymphoplasmacytic infiltration are both important histological features of IgG4-RD [4], which also appear in RA synovium. This study demonstrated that marked IgG4-positive plasma cells infiltrated in rheumatoid synovium and correlated with total synovitis score, inflammatory infiltration subscore, CD3-positive T cells, CD20-positive B cells, or CD38-positive plasma cells. SIgG4 also correlated positively with IgG4-positive plasma cells, but not with total synovitis score or its subscores, maybe due to the small number of patients.

Fibrosis is another vital histological feature of IgG4-RD, especially storiform fibrosis which resembles the spokes of a cartwheel with spindle cells radiating from a center [39]. Our result showed fibrosis was also present in RA synovium. Transforming growing factor (TGF)-*β*, a powerful fibrogenic cytokine, was upregulated and might induce fibrosis in the involved organ(s) of IgG4-RD [20]. Fibrosis of RA synovium may also be induced by TGF-*β* which was also upregulated in RA synovium [40]. However, further studies are needed to elucidate the pathogenetic role of synovial IgG4 and its correlation with fibrosis in RA.

Overall, the role of IgG4 in RA remains elusive and controversial. IgG4 may be an anti-inflammatory molecule for Fab (fragment antigen binding) arm exchange. One IgG4 swap a heavy chain and attached light chain (half-molecule) with a heavy-light chain pair from another IgG molecule, which results in bispecific antibodies without ability of crosslink antigens or form immune complexes [41]. Additionally, Fc (fragment crystallizable) of IgG4 interacted with Fc of other subclasses of IgG to dampen the inflammatory response of these IgG molecules [42]. However, IgG4 may serve as pathogenic autoantibody in certain disease. For instance, for patients with pemphigus foliaceus, an autoimmune blistering skin disease, IgG4 is the predominant antibody against desmoglein 1 and mediates the formation of cutaneous blisters [43, 44]. Thus, further studies are needed to clarify the exact pathogenetic role of IgG4 in RA.

## 5. Conclusion

Our results showed that elevated sIgG4 in RA is common and disproportional to total IgG and RA with elevated sIgG4 may be a specific clinical phenotype with higher disease activity, higher level of autoantibodies, and poor response to MTX + LEF therapy. Further studies will contribute to the elucidation of IgG4 in the pathogenesis of RA.

## Figures and Tables

**Table 1:** Baseline demographic characteristics of all RA patients and untreated RA patients.

Characteristics	All patients	Untreated patients
	(*n* = 136)	(*n* = 41)
Age, years	52 ± 14 (19~86)	53 ± 12 (29~81)
Gender, *n* (%) female	85 (62.5)	24 (58.5)
Disease duration, months	69 ± 74 (1~360)	65 ± 69 (1~264)
28TJC	9 ± 9 (0~28)	12 ± 9 (0~28)
28SJC	7 ± 8 (0~26)	8 ± 7 (0~24)
HAQ score	1.0 ± 0.9 (0.0~3.0)	1.1 ± 0.7 (0.0~3.0)
PtGA	5 ± 3 (0~10)	6 ± 3 (1~10)
PrGA	5 ± 3 (0~10)	6 ± 2 (1~10)
Pain VAS	4 ± 3 (0~10)	5 ± 2 (0~10)
CRP, mg/L	31 ± 38 (0.2~228)	38 ± 32 (1~149)
ESR, mm/h	58 ± 38 (1~148)	71 ± 39 (1~148)
IgE, IU/mL	397 ± 1438 (4~13300)	766 ± 2322 (5~13300)
Eosinophils, 10^9^/L	0.2 ± 0.2 (0.0~0.9)	0.2 ± 0.2 (0.0~0.6)
RF, IU/mL	367 ± 532 (10~2940)	431 ± 560 (10~2590)
Anti-CCP Ab, U/mL	205 ± 224 (2~805)	215 ± 247 (2~805)
DAS28(4)-CRP	4.6 ± 1.8 (1.0~8.2)	5.4 ± 1.4 (2.4~7.7)
Medications before enrollment		
Prednisone	72 (52.9)	—
Methotrexate	60 (44.1)	—
Leflunomide	47 (34.6)	—
Sulfasalazine	12 (8.8)	—
Hydroxychloroquine	17 (12.5)	—
Cyclosporine	1 (0.7)	—
TNF-*α* antagonist^*^	16 (11.8)	—

Data were described with mean ± standard deviation (range) or number (percentage) unless stated otherwise.

28TJC: 28 tender joint counts; 28SJC: 28 swollen joint counts; HAQ: Health Assessment Questionnaire; PtGA: patient global assessment of disease activity; PrGA: provider global assessment of disease activity; VAS: visual analog scales; CRP: C-reactive protein; ESR: erythrocyte sedimentation rate; RF: rheumatoid factor; anti-CCP Ab: anticyclic citrullinated peptide antibody; DAS28: Disease Activity Score with 28-joint counts.

^*^Two (1.5%) patients had received treatment of infliximab, 14 (10.3%) patients had received treatment of Recombinant Human Tumor Necrosis Factor-*α* Receptor IΙ: IgG Fc Fusion Protein for Injection.

**Table 2:** Comparison of autoantibodies of 67 RA patients between normal and elevated serum IgG4 groups.

Autoantibodies	Serum IgG4		*P*
	Elevated group	Normal group	
	(*n* = 28)	(*n* = 39)	
Rheumatoid factor (IU/mL)			
At baseline	529 ± 497	338 ± 493	**0.006**
After treatment^#^	136 ± 145	153 ± 407	**0.011**
Reduction range^*^	393 ± 459	185 ± 380	**0.005**
Anti-CCP antibody (U/mL)			
At baseline	307 ± 238	167 ± 214	**0.006**
After treatment	175 ± 134	101 ± 123	**0.015**
Reduction range	131 ± 190	65 ± 130	0.146

Mann-Whitney rank-sum test was used for comparison. Data were presented as mean ± standard deviation.

^#^Autoantibodies were determined again at 6-month visit.

^*^Reduction range = At baseline − After treatment.
